# The pros and cons of nucleic acid-amplified immunoassays—a comparative study on the quantitation of prostate-specific antigen with and without rolling circle amplification

**DOI:** 10.1007/s00216-024-05357-y

**Published:** 2024-06-07

**Authors:** Mariia Dekaliuk, Zdeněk Farka, Niko Hildebrandt

**Affiliations:** 1 Laboratory of Molecular Assays and Imaging, Institute of Bioorganic Chemistry, Polish Academy of Sciences, 61-704 Poznań, Poland; 2 Laboratoire COBRA, CNRS, INSA Rouen, Université de Rouen Normandie, Normandie Université, Rouen, France; 3 Department of Biochemistry, Faculty of Science, Masaryk University, Kamenice 5, 625 00 Brno, Czech Republic; 4 Department of Engineering Physics, McMaster University, 1280 Main Street West, Hamilton, L8S 4L7 Canada

**Keywords:** ELISA, Diagnostics, PSA, Fluorescence, Terbium, TR-FRET

## Abstract

Integrating isothermal nucleic acid amplification strategies into immunoassays can significantly decrease analytical limits of detection (LODs). On the other hand, an amplification step adds time, complication, reagents, and costs to the assay format. To evaluate the pros and cons in the context of heterogeneous multistep immunoassays, we quantified prostate-specific antigen (PSA) with and without rolling circle amplification (RCA). In addition, we compared time-gated (TG) with continuous-wave (CW) photoluminescence (PL) detection using a terbium complex and a fluorescein dye, respectively. For both direct (non-amplified) and amplified assays, TG PL detection provided circa four- to eightfold lower LODs, illustrating the importance of autofluorescence background suppression even for multi-wash assay formats. Amplified assays required an approximately 2.4 h longer assay time but led to almost 100-fold lower LODs down to 1.3 pg/mL of PSA. Implementation of TG-FRET (using a Tb-Cy5.5 donor–acceptor pair) into the RCA immunoassay resulted in a slightly higher LOD (3.0 pg/mL), but the ratiometric detection format provided important benefits, such as higher reproducibility, lower standard deviations, and multiplexing capability. Overall, our direct comparison demonstrated the importance of biological background suppression even in heterogeneous assays and the potential of using isothermal RCA for strongly decreasing analytical LODs, making such assays viable alternatives to conventional enzyme-linked immunosorbent assays (ELISAs).

**Graphical Abstract:**

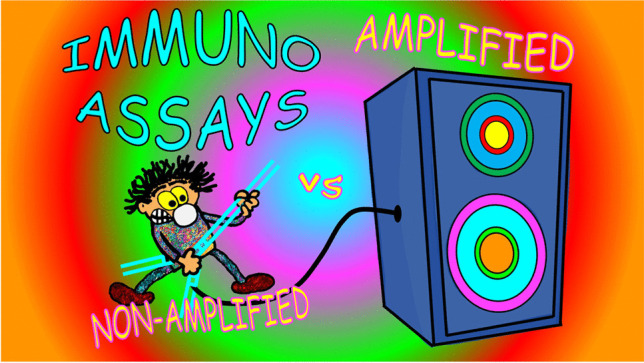

**Supplementary Information:**

The online version contains supplementary material available at 10.1007/s00216-024-05357-y.

## Introduction

Immunoassays have been extensively utilized in research and clinical practice for several decades [1]. They found applications in various fields, including cancer, infection, and cardiovascular disease diagnostics [2–4]. Among the available methods, enzyme-linked immunosorbent assays (ELISAs) [5, 6] stand out as the most commonly used technique due to their high selectivity, sensitivity, reproducibility, and relatively straightforward assay format. With the remarkable affinity of antibodies, ELISA technology can reach limits of detection (LODs) as low as pg/mL concentrations for certain target analytes [7]. Despite their widespread use, there remain challenges in utilizing ELISAs for detecting extremely low target concentrations [8], in particular, for early diagnosis of various diseases or infections and for multiplexing. To enhance the detection sensitivity and versatility of ELISAs, different strategies, such as fluorescence-linked immunosorbent assays (FLISAs) [9, 10], digital ELISAs [11], or microfluidic ELISAs [12], have been proposed. Nucleic acid amplification is another possibility for enhancing the assay performance [13–19]. Rolling circle amplification (RCA) is one of the most commonly applied isothermal amplification techniques [20], and RCA has already been used for the ultra-sensitive detection of target proteins [14, 18, 21, 22]. One prominent example is proximity ligation [23, 24], which has been commercially available for many years [25].

**Fig. 1:**
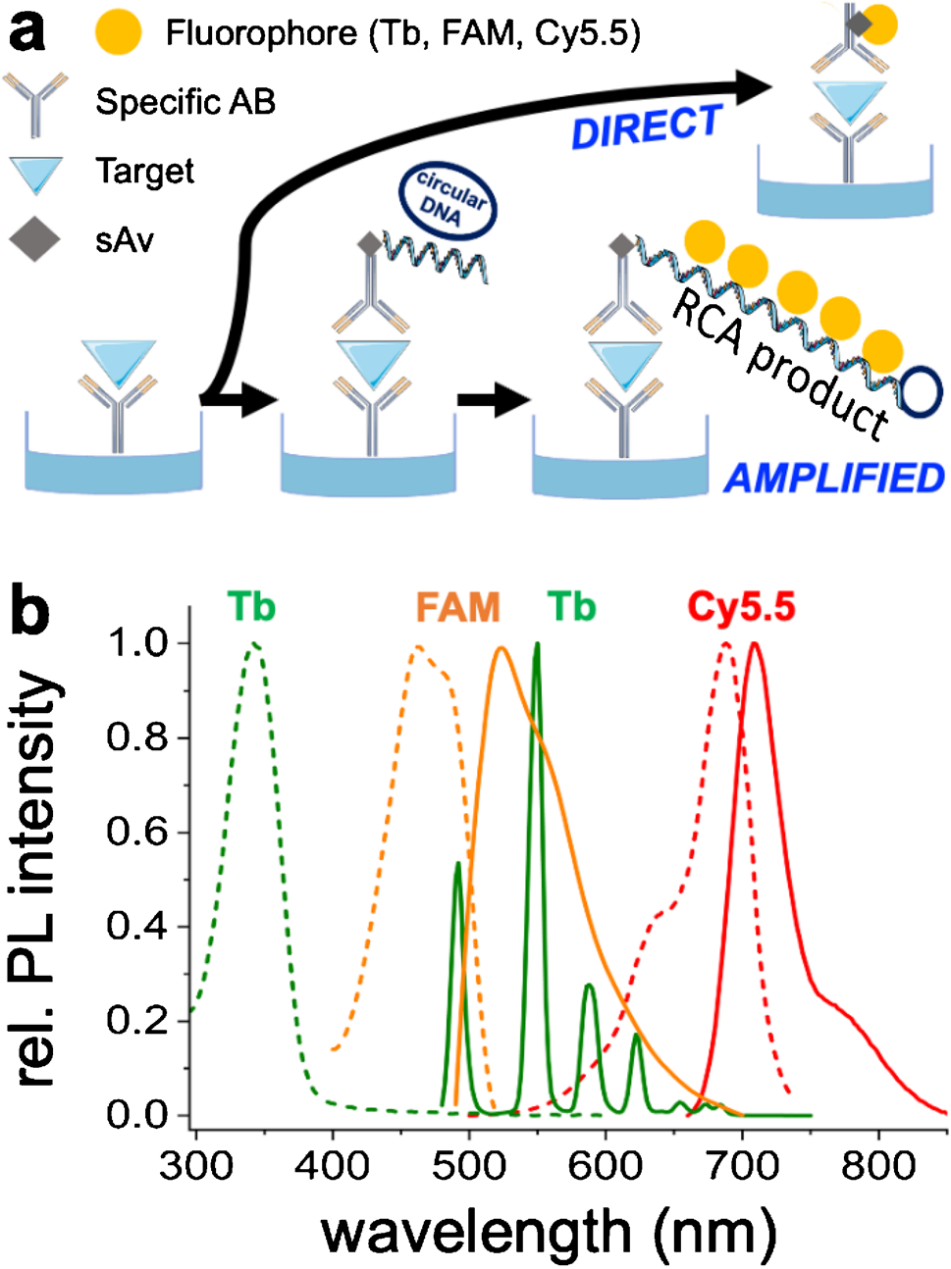
**a** Schematic representation of the direct and amplified immunoassays investigated. Both assay types used 96-well plates coated with PSA-specific capture antibodies (AB). After target incubation and washing, the PSA (bound to the capture antibodies) was recognized by biotinylated detection AB. For the direct approach, fluorophore-labeled streptavidin (sAv) attached to the biotinylated detection AB. For the amplified approach, sAv attached to the biotinylated detection AB, and biotinylated oligonucleotides attached to sAv. The oligonucleotides served as primers for RCA on the circular DNA template, followed by fluorescent DNA-probe labeling of the RCA product. **b** Excitation (dotted) and emission (solid) spectra of the Tb (excitation/emission wavelengths: 342 nm/490, 550, 588, 624 nm), FAM (excitation/emission wavelengths: 460 nm/524 nm), and Cy5.5 (excitation/emission wavelengths: 690 nm/710 nm)

Some immunoassays require low LODs whereas others must be simple and rapid. Thus, from the analytical point of view, it would be very interesting to evaluate the pros and cons of amplified *versus* direct, *i.e.*, non-amplified, assays within the same test format. With this objective in mind, we developed a sandwich FLISA, in which capture antibodies (ABs) were immobilized on 96-well plates, followed by target binding and subsequent incubation with “direct” or “amplified” probe ABs (Fig. 1a). While the ABs in direct detection were labeled with fluorophores, the ABs in amplified assay were labeled with short oligonucleotides that served as specific primer for RCA on a circular DNA template. The amplified RCA product is a long single-stranded (ss) DNA concatemer that can subsequently be labeled with multiple short fluorescent ssDNA probes. Thereby, a single sandwich AB-target-AB binding event can be probed by thousands of fluorophores versus less than 10 fluorophores for the direct assay.

To also investigate the influence of different photoluminescence (PL) detection techniques, we used fluorescein (FAM) as a fluorescent dye for continuous-wave (CW) PL detection, Lumi4-Tb (Tb) as terbium complex for time-resolved or time-gated (TG) PL detection, and the Tb/Cyanine5.5 (Cy5.5) Förster resonance energy transfer (FRET) pair for TG-FRET PL detection (Fig. 1b). Owing to the long luminescence lifetimes of lanthanide complexes, TG and TG-FRET PL detection provide efficient suppression of background signals and concomitant reduction of LODs [26]. Moreover, combining different lanthanide FRET donors with different FRET acceptors can enable efficient multiplexing [27, 28]. In particular, the Tb-Cy5.5 FRET pair was demonstrated to be very useful for RCA-based quantitation of different DNA- and RNA-based biomarkers [29–33].

We selected prostate-specific antigen (PSA) as a proof-of-concept biomarker [34, 35]. The clinical cut-off level for PSA is typically set at 4 ng/mL, with concentrations above this threshold indicating a higher probability of prostate cancer [36]. All assays were measured on a benchtop fluorescence plate reader and provided LODs below 1 ng/mL. Despite the heterogeneous assay format, for which various incubation and separation steps strongly reduce the background signals from unwanted components, the direct TG PL assay (using Tb) showed an approximately fourfold lower LOD than the direct CW PL assay (using FAM). Whereas the amplified assays required additional steps within the assay protocol and approximately 2 h longer time for preparation, their LODs were almost two orders of magnitude lower. The amplified Tb TG PL assay (LOD of circa 1.3 pg/mL) showed an approximately eightfold lower LOD than the amplified FAM CW PL assay (LOD of 10 pg/mL). The amplified TG FRET assay (using the Tb-Cy5.5 FRET pair) had an LOD of 3 pg/mL but provided the capability for multiplexing and reduced washing steps. Overall, our results show that signal amplification in immunoassays is a viable solution to decrease LODs at the cost of increasing assay complexity. Considering that the quantitation of ultra-low target concentrations is not always the most crucial figure of merit for an immunoassay, the actual application must be carefully considered when balancing between ease of use and analytical sensitivity.

## Materials and methods

### Materials

Monoclonal anti-PSA capture antibody (ab403) and PSA (ab78528) were purchased from Abcam (UK). Polyclonal anti-PSA detection antibody (AF1344) was purchased from R&D Systems (USA). Streptavidin (sAv) from *Streptomyces avidinii* (S0677) was purchased from Sigma-Aldrich. Lumi4-Tb-NHS and Lumi4-Tb-sAv (5:1 Tb/streptavidin) were provided by Lumiphore (USA). NHS-fluorescein (46409) and fetal bovine serum (FBS, value, Gibco, A5256701) were purchased from Thermo Fisher. The following buffers were used: carbonate buffer (100 mM NaHCO_3_/Na_2_CO_3_, pH 9.0 at 25 °C), 100 mM HEPES buffer, pH 7.4 at 25 °C, hybridization buffer (20 mM Tris–Cl, 500 mM NaCl, 2 mM MgCl_2_, 0.1% BSA, pH 8.0 at 25 °C), coating buffer (100 mM NaHCO_3_/Na_2_CO_3_, pH 9.0 at 25 °C), phosphate-buffered saline (PBS, 10 mM NaH_2_PO_4_/Na_2_HPO_4_,140 mM NaCl, 3 mM KCl pH 7.4 at 25 °C), assay buffer (10% SuperBlock (Thermo Fisher, 37515) in PBS + 0.01% Tween20), and washing buffer (50 mM NaH_2_PO_4_/Na_2_HPO_4_, 0.01% Tween 20, 0.05% NaN_3_, pH 7.4). All buffer components were purchased from Sigma-Aldrich. Custom oligonucleotides (oligos) were purchased from Eurogentec (Belgium). Primer oligo: 5′-biotin-AAA-AAA-AAA-AAA-AAA-CAC-AGC-TGA-GGA-TAG-GAC-AT; padlock oligo: 5′-phosphate-CTC-AGC-TGT-GTA-ACA-ACA-TGA-AGA-TTG-TAG-GTC-AGA-ACT-CAC-CTG-TTA-GAA-ACT-GTG-AAG-ATC-GCT-TAT-TAT-GTC-CTA-TC, labeling oligos: 5′-NH_2_-C6-TCA-GAA-CTC-ACC-TGT-TAG, 5′-FAM-C6-TCA-GAA-CTC-ACC-TGT-TAG, 5′-Cy5.5-C6-AAA-CTG-TGA-AGA-TCG-CT. Phi29 polymerase, T4 DNA ligase, Taq DNA ligase, ExoI, nuclease-free water, and dNTP mixture were obtained from New England Biolabs (USA). CircLigase ssDNA Ligase was purchased from VWR France.

### Conjugation of the detection polyclonal AB with biotin

Conjugation of the anti-PSA antibody AF1344 with biotin was performed according to the protocol by Hermanson [37]. In brief, 10 mg/mL of biotinamidohexanoic acid *N*-hydroxysuccinimide ester (NHS-LC-biotin) dissolved in anhydrous DMF was added in 15-fold molar excess to the anti-PSA antibody in PBS (0.2 mg/mL) as two aliquots 10 min apart. The reaction was carried out for 30 min at room temperature with mild shaking, followed by incubation at 4 °C overnight. The AB-biotin conjugate was purified to PBS using Amicon Ultra 0.5 mL centrifugal filters 100K (100 kDa MWCO) and stored at 4 °C. The concentration of the purified biotinylated antibody was measured using a Qubit4 fluorometer with a Protein broad range kit.

### Conjugation of sAv with FAM

Conjugation of sAv with FAM was performed according to the protein labeling protocol. NHS-fluorescein (0.1 mg/mL) dissolved in anhydrous DMF was added in 15-fold molar excess to sAv in PBS (10 mg/mL) and mixed well. The reaction was carried out at 4 °C overnight. The sAv-FAM conjugate was purified to PBS using Amicon Ultra 0.5 mL centrifugal filters 50 K (50 kDa MWCO) and stored at 4 °C. The concentration of the purified conjugate and labeling ratio were calculated from absorbance measurements at 280 nm and 493 nm using a BMG SPECTROstar UV–Vis absorption spectrometer as described by Thermo Fisher “labeling with NHS-Fluorescein” protocol. The determined molar FAM-to-sAv ratio was 7:1.

### Conjugation of oligos with Tb

Tb-oligo conjugation was performed following the protocol described previously [32]. Lumi4-Tb-NHS dissolved in anhydrous DMF (8 mM) was added in 16-fold molar excess to amino-functionalized oligonucleotide in 100 mM carbonate buffer at pH 9.0. The mixture was carefully vortexed and incubated at 4 °C overnight. Tb-oligo conjugate was purified three times by 7 K Zeba Spin Desalting columns. The concentration of the purified conjugate and labeling ratio was calculated from absorbance measurement at 260 nm (oligo) and 340 nm (terbium) using a BMG absorption spectrometer; the concentration was 9.5 µM, and the Tb-to-oligo ratio was 1.2:1.

### FLISA with “direct” Tb/FAM labeling

A 96-well black polystyrene microtiter plate (MaxiSorp, high protein binding capacity) was coated with 1 µg/mL of monoclonal anti-PSA AB in coating buffer at 4 °C overnight. All subsequent steps were carried out at room temperature. After four washing steps with 300 µL of washing buffer, the plate was blocked with 300 µL of SuperBlock for 15 min and washed four times. The PSA dilutions were prepared in assay buffer in a concentration range of 0.001 to 100 ng/mL. In each well, 100 µL of the PSA sample was incubated for 1 h. After four washing steps, 100 µL of 1 µg/mL biotinylated detection AB was added to the plate and incubated for 1 h, followed by four washing steps. For the labeling, 100 µL of the 10 µM Tb-sAv or FAM-sAv conjugate was added for 1 h. After four washing steps, 100 µL of hybridization buffer was added, followed by fluorescence measurement using a TECAN SPARK plate reader utilizing the following parameters: Tb, excitation 337 ± 10 nm, emission 550 ± 2.5 nm; integration time 2 ms, lag time 0.1 ms; FAM, excitation 475 ± 10 nm, emission 520 ± 2.5 nm; integration time 40 µs.

The assays were analyzed using relative PL intensities (PL intensity at a given target concentration divided by PL intensity without target) of Tb or FAM:

$$\mathbf{T}\mathbf{b}: rel. PL intensity= \frac{{I}_{Tb(c=x)}}{{I}_{Tb(c=0)}}$$

$$\mathbf{F}\mathbf{A}\mathbf{M}: rel. PL intensity= \frac{{I}_{FAM(c=x)}}{{I}_{FAM(c=0)}}$$

### Preparation of circular template for RCA

The circular template for RCA was prepared from the linear single-stranded padlock hybridized to the primer oligo (using ligase). To evaluate the performance of padlock circularization and its influence on RCA, various ligases and protocols were applied:

- **(i)** Taq DNA ligase: 1.46 nmol of biotinylated primer oligo, 1.46 nmol of padlock oligo, and 400 U Taq DNA ligase were mixed in 50 µL of adjusted Taq DNA ligase buffer (20 mM Tris–HCl, 25 mM potassium acetate, 10 mM magnesium acetate, 1 mM nicotinamide adenine dinucleotide (NAD 1), 10 mM dithiothreitol (DTT), 0.1% Triton X-100, 0.25 M NaCl, 0.4 mM ATP) and incubated at 37 °C for 45 min. After incubation, the resulting mixture could be used directly or stored at − 20 °C.
- **(ii)** Taq DNA ligase + ExoI: 1.46 nmol of biotinylated primer oligo, 1.46 nmol of padlock oligo, and 400 U Taq DNA ligase were mixed in 50 uL of adjusted Taq DNA ligase buffer and incubated at 37 °C for 45 min. Then, 2U mL^−1^ of ExoI were added to digest the linear DNA residue and incubated for 30 min at 37 °C with the following inactivation for 20 min at 37 °C. Afterward, the resulting mixture could be used directly or stored at − 20 °C.
- **(iii)** T4 DNA ligase: 1.46 nmol of biotinylated primer oligo, 1.46 nmol of padlock oligo, and 400 U T4 DNA ligase were mixed in 50 µL of adjusted Taq DNA ligase buffer and incubated at 37 °C for 45 min. After inactivation at 65 °C for 10 min, the resulting mixture could be used directly or stored at − 20 °C.
- **(iv)** T4 DNA ligase + ExoI: 1.46 nmol of biotinylated primer oligo, 1.46 nmol of padlock oligo, and 400 U T4 DNA ligase were mixed in 50 µL of adjusted Taq DNA ligase buffer and incubated at 37 °C for 45 min. After inactivation at 65 °C for 10 min, 2U mL^−1^ of ExoI were added to digest the linear DNA residue and incubated for 30 min at 37 °C, with the following inactivation for 20 min at 37 °C. The resulting mixture could be used directly or stored at − 20 °C.
- **(v)** CircLigase ssDNA ligase kit: 2.5 nmol of padlock oligo, 400 U CircLigase, 0.1 mM ATP, 2.5 mM MgCl_2_ mixed in 50 µL 1 × CircLigase reaction buffer were incubated at 60 °C for 60 min, followed by inactivation at 80 °C for 20 min. Then, 2 U mL^−1^ of ExoI were added to digest the linear DNA residue and incubated for 30 min at 37 °C, with following inactivation for 20 min at 37 °C. The resulting mixture could be used directly or stored at − 20 °C.

### Validation of circular template for RCA

Validation of the RCA performance (using the different approaches i to v from above) was performed using a fixed design strategy: A 96-well black polystyrene microtiter plate (MaxiSorp, high protein binding capacity) was coated with 1 µg/mL of monoclonal anti-PSA AB in coating buffer at 4 °C overnight. All subsequent steps were carried out at room temperature (RT). After four washing steps with 300 µL of washing buffer, the microtiter plate was blocked with 300 µL of SuperBlock for 15 min and washed four times. The PSA dilutions were prepared in assay buffer in concentrations of 0.05, 0.5, and 1 ng/mL. For each well, 100 µL of the PSA sample was added and incubated for 1 h. Then, after three washing steps with washing buffer, 100 µL of 1 µg/mL of detection AB was added to the plate and incubated for 1 h. After four washing steps, the microtiter plate was incubated with 100 µL of 1 µg/mL of sAv in assay buffer for 15 min. After one washing step, 75 µL of 50 nM target probe in assay buffer was added, incubated for 15 min at RT, and washed once. Then, 75 µL of 50 nM circulated probe (described above) in assay buffer was added, incubated for 30 min at RT, and washed gently twice. Next, 50 µL of polymerization mixture was added, 0.8 U µL^−1^ phi29 polymerase, 0.25 µg/mL BSA, and 0.5 mM dNTP in 1 × phi29 polymerase buffer, and incubated for 90 min at RT. After one washing step, 50 µL of 200 nM Tb-labeled oligo labeling probe in hybridization buffer was added and incubated for 30 min at RT. After three washing steps, 100 µL of hybridization buffer was added, followed by fluorescence measurement using a TECAN SPARK plate reader utilizing the following parameters: Tb, excitation 337 ± 10 nm, emission 550 ± 2.5 nm; integration time 2 ms, lag time 0.1 ms.

We validated three different ligases to circularize the padlock probe, following the protocols described above. The T4DNA and Taq DNA ligases require previous annealing with the biotinylated primer oligonucleotide, while the CircLigase kit can produce a circular template without needing a primer. To remove non-circularized single-stranded oligos from the RCA templates, we applied Exonuclease I (ExoI) to some samples. The comparison of RCA performance is shown in Fig. S1. Taq DNA ligase demonstrated the lowest RCA performance, regardless of the use of ExoI. The commercial CircLigase kit and T4 DNA ligase assisted with ExoI purification and demonstrated relatively similar performance. However, it was observed that the application of the CircLigase kit, T4 DNA Ligase, and Taq DNA Ligase (without ExoI treatment) exhibited similarities in decreasing the efficiency of RCA at higher protein target concentrations (1 ng mL^−1^). Such an effect is potentially due to less efficient circular template production, resulting in a lack of RCA templates in the amplification system. In contrast, for the T4 DNA ligase + ExoI system, a linear increase in PL intensity was observed over the used concentration range, confirming efficient assay performance. Thus, this circularization approach (protocol iv) was selected for all further amplification assays conducted in our study.

### FLISA with “amplified” Tb/FAM/FRET labeling

Regardless of the labeling strategy, all amplification-based assays were carried out using the same protocol except for the final labeling step. A 96-well black (MaxiSorp, high protein binding capacity) microtiter plate was coated with 1 µg/mL of monoclonal anti-PSA AB in coating buffer at 4 °C overnight. All subsequent steps were carried out at RT. After three washing steps (300 µL of washing buffer for all the following steps), the plate was blocked with 300 µL of SuperBlock for 15 min. After three washing steps, 100 µL of PSA dilutions in a concentration range of 0.001 to 100 ng/mL in assay buffer were added and incubated for 1 h. Then, after three washing steps, 100 µL of 1 µg/mL of the detection AB was added to the plate and incubated 1 h. After three washing steps, the microtiter plate was incubated with 100 µL of 1 µg/mL of sAv in assay buffer for 15 min. After one washing step, 75 µL of 50 nM target probe in assay buffer was added, incubated for 15 min at RT, and washed once. Then, 75 µL of 50 nM circulated probe (protocol “iv” T4 DNA ligase + ExoI) in assay buffer was added, incubated for 30 min at RT, and washed gently twice. Next, 50 µL of polymerization mixture was added, 0.8 U µL^−1^ phi29 polymerase, 0.25 µg/mL BSA, 0.5 mM dNTP in 1 × phi29 polymerase buffer, and incubated for 90 min at RT. After one washing step, 75 µL of (a) 200 nM Tb-oligo, (b) 200 nM FAM-oligo, or (c) a mixture of 200 nM Tb-oligo and 200 nM Cy5.5-oligo in hybridization buffer was added and incubated for 30 min at RT. After three washing steps, 100 µL of hybridization buffer was added, followed by fluorescence measurement using a TECAN SPARK plate reader. Note that for (c), the last washing step can be eliminated because FRET can only occur on the RCA product and free Tb-oligos and Cy5.5-oligos do not need to be eliminated. The following measurement parameters were utilized: Tb, excitation 337 ± 10 nm, emission 550 ± 3.75 nm; integration time 2 ms, lag time 0.1 ms; FAM, excitation 475 ± 10 nm, emission 520 ± 2.5 nm. The single fluorophore (Tb or FAM) assays were analyzed using relative PL intensities. FRET(Tb-Cy5.5): excitation 337 ± 10 nm, emission (Tb) 494 ± 2.5 nm, emission (Cy5.5) 716 ± 5 nm; integration time 2 ms, lag time 0.1 ms. The Tb-Cy5.5 assays were analyzed using the relative FRET ratio of the TG PL intensity of the Cy5.5 acceptor (at 716 nm) and the TG PL intensity of the Tb donor (at 494 nm):

$$\begin{array}{cc}\mathbf{F}\mathbf{R}\mathbf{E}\mathbf{T}:FRET ratio= \frac{{I}_{Cy5.5(c=x)}}{{I}_{Tb(c=x)}};& rel. FRET ratio= \frac{FRET ratio (c = x)}{FRET ratio (c=0)}\end{array}$$

## Results and discussion

### Optical properties of the labeling probes

To compare similar PL wavelengths (Fig. 1b), the fluorescent dye fluorescein (FAM) and the lanthanide complex Lumi4-Tb (Tb) were selected as CW and TG PL labeling probes, respectively. These probes were conjugated with streptavidin (sAv) for the direct and with short oligos for the amplified assays. The maximum emission/excitation/absorptivity values were 524 nm/460 nm/70000 M^−1^ cm^−1^ for FAM and 550 nm/342 nm/26000 M^−1^ cm^−1^ for Tb. For the amplified TG-RCA-FRET assays, Tb was further combined with Cyanine5.5 (Cy5.5) as a FRET donor–acceptor pair. The spectral overlap of Tb emission and Cy5.5 absorption (Fig. 1b) resulted in a Förster distance *R*_0_ (Tb-Cy5.5) of 5.8 ± 0.2 nm [33]. Owing to the red acceptor emission beyond the Tb PL, the Tb-Cy5.5 FRET pair provides very efficient DNA or RNA sensing performance [29, 33]. In principle, other lanthanides (e.g., europium) can also be used as FRET donors for nucleic acid sensing [32]. Moreover, a combination with different acceptor dyes can enable both spectral and temporal multiplexing [29].

### Direct PSA immunoassay

**Fig. 2:**
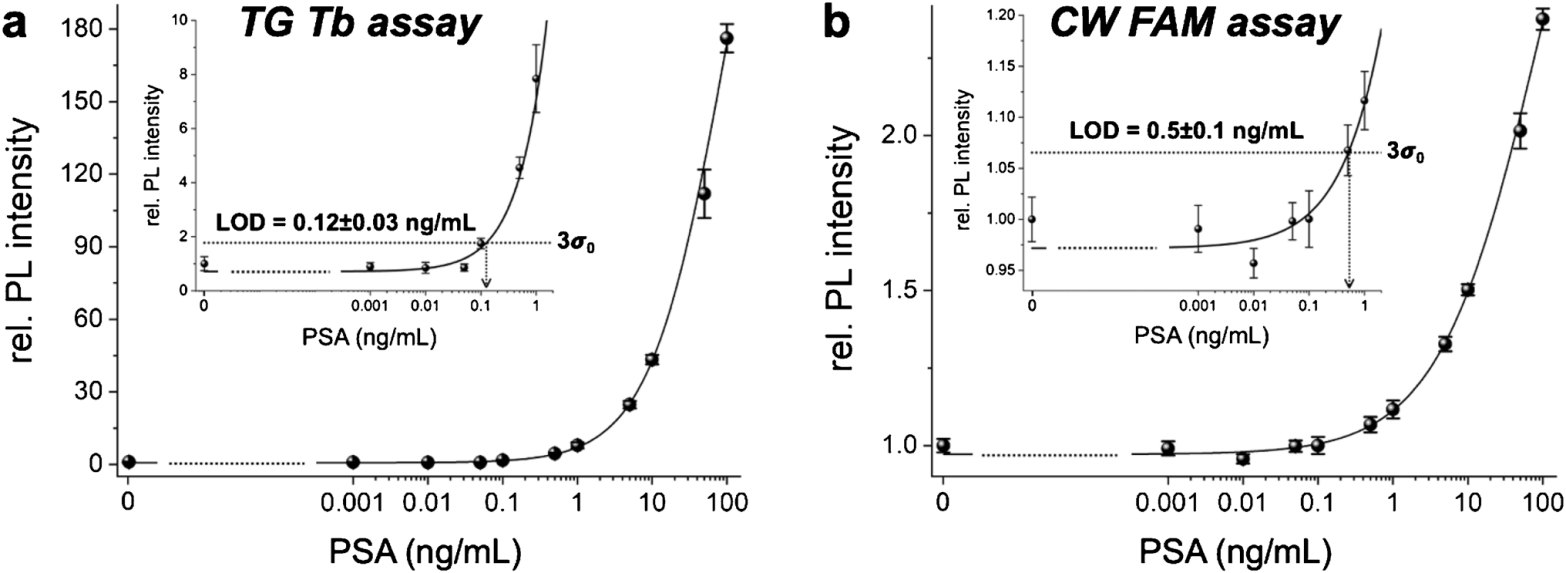
FLISA calibration curves for the direct TG PL (**a**) and CW PL (**b**) PSA assays. The curves between the blank (0 ng/mL) and the lowest measured concentration (0.001 ng/mL) were estimated and are shown as dotted lines. Insets show a magnified view of the lower concentration range. The LODs were evaluated corresponding to three times the standard deviation of blank, i.e., 3σ_0_, above the background, as shown by the dotted lines that cross the calibration curves. Error bars correspond to the standard deviations from three independent measurements (*n* = 3) for all target concentrations

While the suppression of autofluorescence background in immunoassays and other biosensing approaches via TG PL detection (including TG FRET) with long-luminescent lanthanide complexes has been well-known for many decades [1, 26, 38, 39], it cannot be automatically assumed that a TG PL assay is more sensitive than a CW PL assay. The detection performance of an immunoassay is always dependent on the signal-to-background ratio, and both a lower background for TG PL detection (vs. CW detection) and a higher brightness for fluorescent dyes (vs. lanthanide complexes) can lead to higher signal-to-background ratios. We, therefore, compared two direct FLISAs, which used Tb with TG PL detection and FAM with CW PL detection. The assays consisted of several incubation and washing steps, in which sandwich immunocomplexes between immobilized capture AB, PSA, and detection AB were quantified via PL detection of Tb-sAv or FAM-sAv bioconjugates that bound to the biotinylated detection ABs (Fig. 1a). The calibration curves (Fig. 2) showed excellent analytical performance over the selected PSA concentration range from 0.001 to 100 ng/mL. Although both direct FLISAs provided LODs below the clinical cut-off of 4 ng/mL, the Tb-based TG PL assay showed approximately fourfold lower LODs, higher precision (lower standard deviations), and higher accuracy (better fit to the calibration curve). Because the plate reader measurement parameters differed for the distinct assays (adjusted to obtain sufficient PL intensities at low concentrations and thereby low LODs), one cannot directly compare the assay sensitivities (slopes of the calibration curves). Despite the numerous washing steps, the ABs, the PSA, and the hybridization buffer (containing 0.1% BSA) exhibited a significant autofluorescence background. This resulted in higher signal-to-background ratios for the Tb-based TG PL assays (background suppression via TG PL detection) and thus a significantly better assay performance compared to the FAM-based CW assays, in which the background could not be efficiently suppressed. The LOD of 0.12 ng/mL achieved with the TG PL assay is more than one order of magnitude below the threshold of 4 ng/mL but still significantly higher (around one order of magnitude) than most commercial ELISAs for PSA (Supporting Table S1) or nanoparticle-based approaches [40].

### Amplified PSA detection strategy

To investigate the possibility of decreasing LODs and potentially also increasing the assay sensitivity via isothermal nucleic acid amplification, we implemented RCA into the FLISA approach. Within the amplified assay, AB-PSA-AB sandwich immunocomplex formation was the same as for the direct assay, with the only exception of the lowest PSA concentration tested (0.1 pg/mL, i.e., tenfold lower than in the direct assays). The main difference of the RCA-FLISA was the PL probe labeling procedure (Fig. 1a). First, sAv was added to bind to the biotinylated detection AB, followed by the addition of a biotinylated primer oligo, such that sAv was bridging the AB-DNA assembly. RCA was initiated by the addition of the circular DNA template, Phi29 polymerase, and dNTP mixture, such that the primer oligo could hybridize to the circular DNA and thereby serve as a primer for Phi29 to synthesize new DNA around the circular template for 90 min. The resulting RCA product, a long ssDNA concatemer, was then labeled via hybridization with many short luminescent DNA probes. In addition to Tb and FAM probes (similar to the direct assay), we also investigated using Tb-DNA and Cy5.5-DNA FRET probes. This approach had two potential advantages, namely the avoidance of the final washing step to remove the luminescent DNA probes (because the distance-dependent FRET between Tb and Cy5.5 can only occur within the RCA product and not free in solution) and the use of ratiometric PL detection (Cy5.5/Tb PL intensity ratio) for lower deviations.

**Fig. 3:**
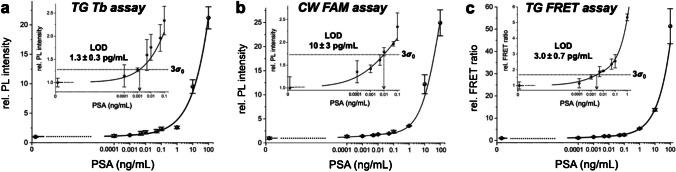
FLISA calibration curves for the amplified TG PL (**a**), CW PL (**b**), and TG FRET (**c**) PSA assays. The curves between the blank (0 ng/mL) and the lowest measured concentration (0.0001 ng/mL) were estimated and are shown as dotted lines. Insets show a magnified view of the lower concentration range. The LODs were evaluated corresponding to three times the standard deviation of blanks, i.e., 3σ_0_, above the background, as shown by the dotted lines that cross the calibration curves. Error bars correspond to the standard deviations from three independent measurements (*n* = 3) for all target concentrations

The assay calibration curves (Fig. 3) revealed that the additional RCA step, which extended the assay procedure by approximately 2 h, reduced the LODs by circa two orders of magnitude. The Tb-based TG PL RCA assay exhibited approximately eight-fold lower LODs compared to the FAM-based CW assay. However, the precision (standard deviations) and accuracy (fit to the calibration curve) were not significantly different between the two detection approaches. Because RCA strongly amplifies the signal and the background is increased to a much lower extent, the similarities in those assay performance parameters are understandable. Only at very low target concentrations, for which the signals are extremely low, the background suppression of TG PL detection becomes advantageous and, thus, enables to achieve lower LODs. TG RCA-FRET led to a slightly higher LOD (around twofold) compared to TG RCA PL detection because the FRET step from Tb to Cy5.5 resulted in splitting of the overall energy over the Tb donor and the Cy5.5 acceptor and a concomitant lower total signal. However, the ratiometric detection (FRET ratio, i.e., acceptor-to-donor TG PL intensity ratio) avoided the final washing step and resulted in significantly higher precision (standard deviations) and accuracy (fit to the calibration curve). Moreover, as previously demonstrated for both protein and nucleic acid detection, the TG FRET approach allows for multiplexed detection using a single Tb donor and different dye or quantum dot acceptors [27]. Overall, the LODs for PSA were all approximately three orders of magnitude below the clinical cut-off concentration. Although some RCA-amplified immunoassays can reach even lower LODs (Supporting Table S2), the circa 100-fold LOD decrease compared to the direct assays and the possibility of ratiometric FRET detection and multiplexing capability demonstrated the high assay performance of the RCA-amplified FLISAs.

Another important issue in immunoassays is selectivity. The commercial antibodies used in our study have been previously utilized in PSA immunoassays and, thus, were already optimized for selectivity. In our heterogeneous assays, AB-PSA recognition occurs before the washing and amplification steps, and since the focus of our study was on the impact of this final amplification versus no amplification, we did not consider selectivity as highly relevant for our comparative analysis. Nevertheless, we recognize that selectivity may ultimately differ between the two assay formats, and for a complete assay development, additional validation of selectivity is recommended.

**Fig. 4:**
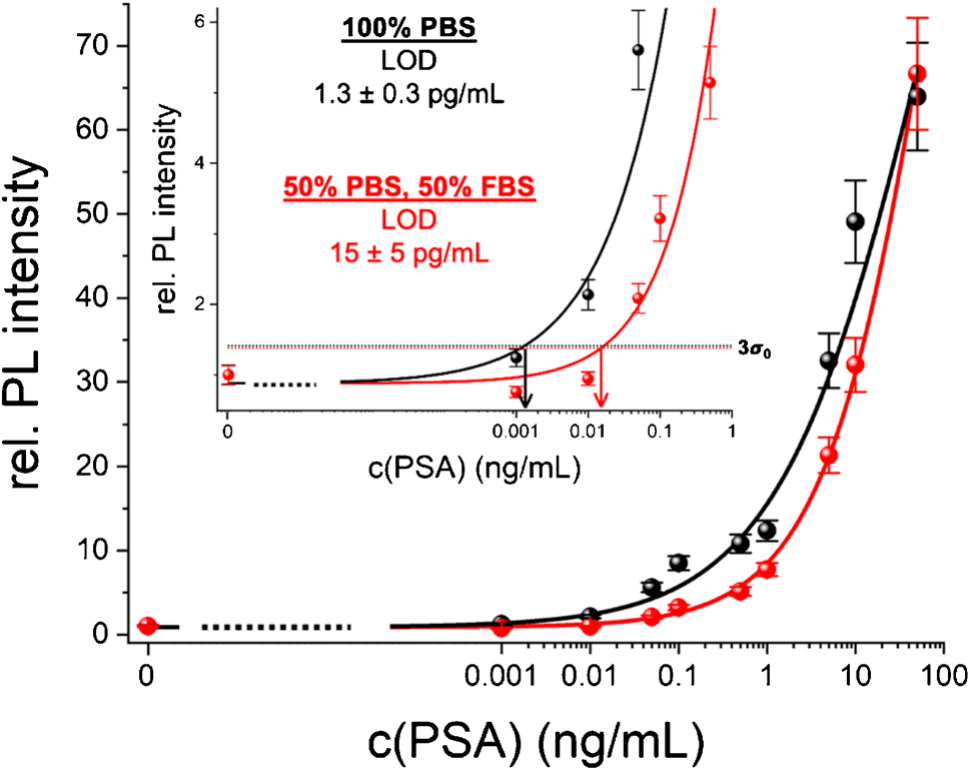
FLISA calibration curves for the amplified TG PL PSA assays with PSA in PBS (black) and 50% FBS (red). The curves between the blank (0 ng/mL) and the lowest measured concentration (0.001 ng/mL) were estimated and are shown as dotted lines. Insets show a magnified view of the lower concentration range. The LODs were evaluated corresponding to three times the standard deviation of blanks, i.e., 3σ_0_, above the background, as shown by the dotted lines that cross the calibration curves. Error bars correspond to the standard deviations from three independent measurements (*n* = 3) for all target concentrations

To demonstrate the compatibility with real-life testing in more complex media than our assay buffer, we performed the TG PL FLISA for PSA samples in phosphate-buffered saline (PBS) and a 1:1 mixture of PBS and fetal bovine serum (FBS) (Fig. 4). Considering that serum components can result in significant non-specific binding, which can lead to a considerable reduction of assay performance, we did not use lower dilutions. The assay performance in PBS was quite similar (LOD of 1.3 pg/mL) to the one in assay buffer, which was expectable, considering that PBS is also a standard immunoassay buffer. However, despite the many washing steps that follow the binding of PSA, the binding of the detection antibody, the binding of sAv, and the probe oligo and the hybridization of Tb-DNA probes, the assay in FBS showed a lower performance with an approximately tenfold higher LOD (15 pg/mL). This result confirms that serum components still influence the assay performance and that non-specific binding cannot be completely eliminated, even via rigorous washing. Nevertheless, even in the samples of 50% serum, low LODs in the pg/mL range could be achieved without diluting the sample too extensively.

## Conclusions

Not taking into account the overnight coating of the microtiter plates with the capture AB (which was the same for both assay types), the total assay times were 3.75 h for the direct and 6.15 h for the amplified immunoassay. Considering the approximately 100-fold improvement in the LOD, the extension of 2.4 h appears acceptable. However, when lower LODs are not required, the additional time, reagents, and costs that come with the amplification should be avoided. Overall, the assay times are similar to other heterogeneous assays (Table S1), which typically range from around 1.5 to 5 h. Decreasing the LOD by two orders of magnitude is relevant especially for clinical targets that require quantitation at ultra-low concentrations, and the compatibility with serum samples showed that the amplified assays are potentially applicable to real-life clinical samples. The combination with TG-FRET may provide the additional benefits of high reproducibility and low standard deviations because of the ratiometric detection format and multiplexing via the application of one Tb donor and different dye acceptors on the RCA product. Importantly, we did not only compare direct versus amplified assays but also different detection methods, namely TG (using lanthanides) and CW (using dyes) PL detection as well as TG-FRET (combining lanthanide donors and dye acceptors). This comparison illustrated the importance of efficient background suppression even for heterogeneous assay formats with multiple washing steps because TG PL and TG FRET were significantly more sensitive (i.e., providing lower LODs) than CW PL. Considering the key pros and cons of both the direct and the amplified immunoassay approaches (Fig. S2), both methods hold significant potential as alternatives to conventional ELISA assays and for future development of clinical and other sensing applications.

## Supplementary Information

Below is the link to the electronic supplementary material.

Supplementary file1 (PDF 420 KB)

## Abbreviations

LOD: Limit of detection
PSA: Prostate-specific antigen
RCA: Rolling circle amplification
TG: Time-gated
CW: Continuous-wave
PL: Photoluminescence
FRET: Förster resonance energy transfer
ELISA: Enzyme-linked immunosorbent assay
FLISA: Fluorescence-linked immunosorbent assay
AB: Antibody
ss: Single-stranded
