# Supplementary material for: The pros and cons of nucleic acid-amplified immunoassays—a comparative study on the quantitation of prostate-specific antigen with and without rolling circle amplification
Source: Anal Bioanal Chem. 2024 Jun 7;416(30):7285–94. doi: 10.1007/s00216-024-05357-y (PMC11584466; doi:10.1007/s00216-024-05357-y)
Supplement: Supplementary file 1 — Supplementary file1 (PDF 420 KB) [file 216_2024_5357_MOESM1_ESM.pdf]

Supplementary Information

The pros and cons of nucleic-acid-amplified immunoassays – A comparative study on the quantitation of prostate-specific antigen with and without rolling circle amplification

Mariia Dekaliuk,[1]* Zdeněk Farka,[2] and Niko Hildebrandt[3]*

[1] Laboratory of Molecular Assays and Imaging, Institute of Bioorganic Chemistry, Polish Academy of Sciences, 61-704 Poznań, Poland.

[2] Department of Biochemistry, Faculty of Science, Masaryk University, Kamenice 5, 625 00 Brno, Czech Republic.

[3] McMaster University, Department of Engineering Physics, 1280 Main Street West, Hamilton, L8S 4L7, Canada.

* Corresponding authors: mdekaliuk@ibch.poznan.pl; hildebrandt@mcmaster.ca

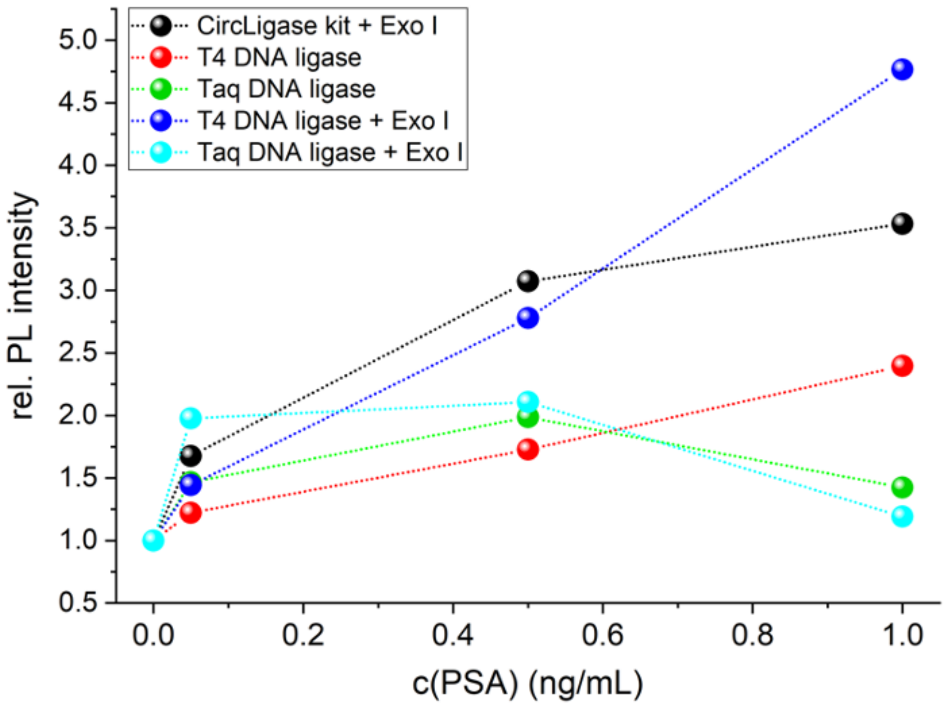

Fig. S1 Influence of different padlock circularization approaches (RCA template formation) on the amplified TG Tb assay performance.

Table S1. List of selected commercial ELISA assays for PSA detection.

Company/ catalog no.	LOD	Reference
Abcam ab264615	8 pg mL^{-1}	https://www.abcam.com/en-pl/products/elisa-kits/human-psa-elisa-kit-ab264615
R&D Systems DKK300	0.069 ng mL^{-1}	https://www.bio-techne.com/p/elisa-kits/human-kallikrein-3-psa-quantikine-elisa-kit_dkk300
Roche Diagnostics Elecsys total PSA	0.01 ng mL^{-1}	https://elabdoc-prod.roche.com/eLD/web/pi/en/products/CPS_000522
Thermo Fisher Scientific EHKLK3T	8 pg mL^{-1}	https://www.thermofisher.com/elisa/product/PSA-Total-KLK3-Human-ELISA-Kit/EHKLK3T
ThermoFisher BRAHMS	0.04 ng mL^{-1}	https://www.brahms.de/en-gb/products/oncology.html

Table S2. Comparison of selected protein detection studies using isothermal rolling circle amplification (RCA) combined with immunoassays.

Method, assay surface	LOD	Detection, probes	Multiplex	Ref.
iRCA-SBA, beads	0.66 pg mL^{-1}	Fluorescence, FITC	Yes	[1]
Electrochemical biosensor, magnetic NPs	22.3 fM (~0.7 pg mL^{-1})	Fluorescence, Au NPs	No	[2]
RCA immunoassay, magnetic NPs	8.3 aM (~1.25 fg mL^{-1})	Fluorescence, SYBR Green I	No	[3]
iRCA, microspots	0.1 pg mL^{-1}	Fluorescence, Cy3	No	[4]
RELISA, plate	2.82 pg mL^{-1}	Absorption, HRP	No	[5]
Cascade fluorescence signal amplification strategy, plate	0.9 fM (~0.15 pg mL^{-1})	Fluorescence, SYBR Green I	No	[6]
NRCA, plate	5.5 fg L^{-1} (~5.5 ag mL^{-1})	Fluorescence, SYBR Green I	No	[7]
PLARCA, plate	0.02 pg mL^{-1}	Absorption, HRP	No	[8]
Protein-responsive FRET immunoassay, in bulk	0.23 pg mL^{-1}	Fluorescence, FRET FAM to TAMRA	No	[9]

HRP: horseradish peroxidase

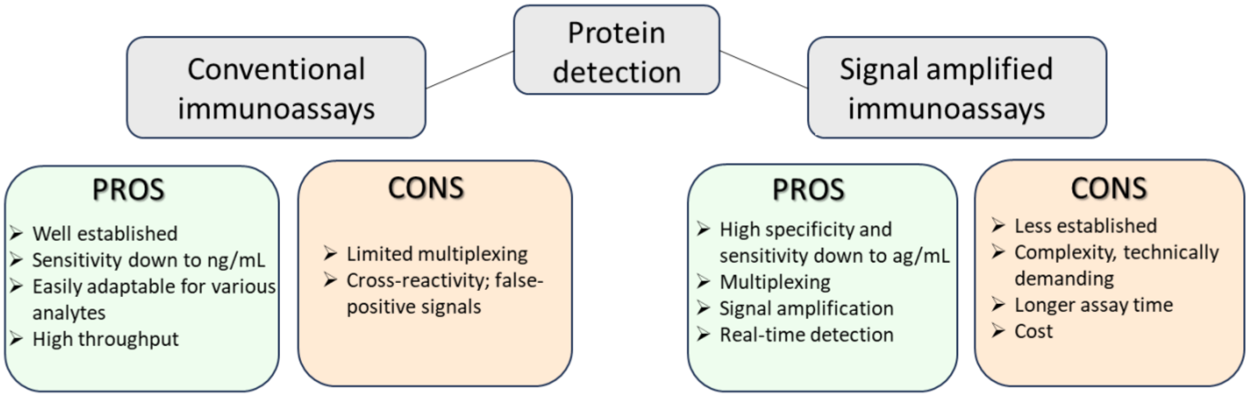

Fig. S2 Key pros and cons of conventional and amplified immunoassays for protein detection.

References

1. Gao M, Lian H, Yu L, Gong M, Ma L, Zhou Y, Yu M, Yan X Rolling circle amplification integrated with suspension bead array for ultrasensitive multiplex immunodetection of tumor markers. Anal Chim Acta. 2019;1048:75–84. https://doi.org/10.1016/j.aca.2018.10.001
2. Lee CY, Fan HT, Hsieh YZ Disposable aptasensor combining functional magnetic nanoparticles with rolling circle amplification for the detection of prostate-specific antigen. Sens Actuators B Chem. 2018;255:341–347. https://doi.org/10.1016/j.snb.2017.08.061
3. Xue Q, Wang L, Jiang W. A versatile platform for highly sensitive detection of protein: DNA enriching magnetic nanoparticles based rolling circle amplification immunoassay. Chemical Communications. 2012;48:3930–3932. https://doi.org/10.1039/c2cc18007c
4. Schweitzer B, Wiltshire S, Lambert J, O'Malley S, Kukanskis K, Zhu Z, Kingsmore SF, Lizardi PM, Ward DC. Immunoassays with rolling circle DNA amplification: A versatile platform for ultrasensitive antigen detection. PNAS. 2000;97:10113–10119. https://doi.org/10.1073/pnas.170237197
5. You M, Peng P, Xue Z, Tong H, He W, Mao P, Liu Q, Yao C, Xu F. A fast and ultrasensitive ELISA based on rolling circle amplification. Analyst. 2021;146:2871–2877. https://doi.org/10.1039/d1an00355k
6. Xue Q, Wang Z, Wang L, Jiang W. Sensitive detection of proteins using assembled cascade fluorescent DNA nanotags based on rolling circle amplification. Bioconjug Chem. 2012;23:734–739. https://doi.org/10.1021/bc200537g
7. Feng C, Bo B, Mao X, Shi H, Zhu X, Li G. From interface to solution: Integrating immunoassay with netlike rolling circle amplification for ultrasensitive detection of tumor biomarker. Theranostics. 2017;7:31–39. https://doi.org/10.7150/thno.16671
8. Ebai T, Souza de Oliveira FMS, Löf L, Wik L, Schweiger C, Larsson A, Keilholtz U, Haybaeck J, Landegren U, Kamali-Moghaddam M. Analytically sensitive protein detection in microtiter plates by proximity ligation with rolling circle amplification. Clin Chem. 2017;63:1497–1505. https://doi.org/10.1373/clinchem.2017.271833
9. Yang W, Shen Y, Zhang D, Xu W. Protein-responsive rolling circle amplification as a tandem template to drive amplified transduction of fluorescence signal probes for highly sensitive immunoassay. Chemical Communications. 2018;54:10195–10198. https://doi.org/10.1039/C8CC04395G
